# An Endophytic Fungi-Based Biostimulant Modulates Volatile and Non-Volatile Secondary Metabolites and Yield of Greenhouse Basil (*Ocimum basilicum* L.) through Variable Mechanisms Dependent on Salinity Stress Level

**DOI:** 10.3390/pathogens10070797

**Published:** 2021-06-23

**Authors:** Sergio Saia, Giandomenico Corrado, Paola Vitaglione, Giuseppe Colla, Paolo Bonini, Maria Giordano, Emilio Di Stasio, Giampaolo Raimondi, Raffaele Sacchi, Youssef Rouphael

**Affiliations:** 1 Department Veterinary Sciences, University of Pisa, via delle Piagge 2, 56129 Pisa, Italy; sergio.saia@unipi.it; 2 Department of Agricultural Sciences, University of Naples Federico II, 80055 Naples, Italy; giandomenico.corrado@unina.it (G.C.); paola.vitaglione@unina.it (P.V.); maria.giordano@unina.it (M.G.); emiliodistasio@gmail.com (E.D.S.); giampaolo.raimondi@unina.it (G.R.); raffaele.sacchi@unina.it (R.S.); 3 Department of Agriculture and Forest Sciences, University of Tuscia, 01100 Viterbo, Italy; giucolla@unitus.it; 4 NGAlab, La Riera de Gaia, 43762 Tarragona, Spain; pb@ngalab.com

**Keywords:** *Funneliformis mosseae*, medicinal and aromatic plants, polyphenols, *Rhizoglomus irregular*, *Trichoderma koningii*, volatile organic compounds

## Abstract

Salinity in water and soil is one of the major environmental factors limiting the productivity of agronomic and horticultural crops. In basil (*Ocimum basilicum* L., Lamiaceae) and other *Ocimum* species, information on the plant response to mild salinity levels, often induced by the irrigation or fertigation systems, is scarce. In the present work, we tested the effectiveness of a microbial-based biostimulant containing two strains of arbuscular mycorrhiza fungi (AMF) and *Trichoderma koningii* in sustaining greenhouse basil yield traits, subjected to two mild salinity stresses (25 mM [low] and 50 mM [high] modulated by augmenting the fertigation osmotic potential with NaCl) compared to a non-stressed control. The impact of salinity stress was further appraised in terms of plant physiology, morphological ontogenesis and composition in polyphenols and volatile organic compounds (VOC). As expected, increasing the salinity of the solution strongly depressed the plant yield, nutrient uptake and concentration, reduced photosynthetic activity and leaf water potential, increased the Na and Cl and induced the accumulation of polyphenols. In addition, it decreased the concentration of Eucalyptol and β-Linalool, two of its main essential oil constituents. Irrespective of the salinity stress level, the multispecies inoculum strongly benefited plant growth, leaf number and area, and the accumulation of Ca, Mg, B, p-coumaric and chicoric acids, while it reduced nitrate and Cl concentrations in the shoots and affected the concentration of some minor VOC constituents. The benefits derived from the inoculum in term of yield and quality harnessed different mechanisms depending on the degree of stress. under low-stress conditions, the inoculum directly stimulated the photosynthetic activity after an increase of the Fe and Mn availability for the plants and induced the accumulation of caffeic and rosmarinic acids. under high stress conditions, the inoculum mostly acted directly on the sequestration of Na and the increase of P availability for the plant, moreover it stimulated the accumulation of polyphenols, especially of ferulic and chicoric acids and quercetin-rutinoside in the shoots. Notably, the inoculum did not affect the VOC composition, thus suggesting that its activity did not interact with the essential oil biosynthesis. These results clearly indicate that beneficial inocula constitute a valuable tool for sustaining yield and improving or sustaining quality under suboptimal water quality conditions imposing low salinity stress on horticultural crops.

## 1. Introduction

Medicinal and aromatic plants (MAPs) encompass a wide range of species grown in greenhouses or open-field conditions. These plants are employed in the food sector, land reclamation and bioremediation. Depending on their multiple uses, MAPS are considered ornamental, culinary or industrial herbs, for pharmaceutical and cosmetic products and essential oils [1,2]. Despite their large cultural and economic importance, information on the variation of yield and quality traits according to the environment and agronomic management is limited. It is generally assumed that MAPs have been little improved to withstand biotic or abiotic stresses, despite adverse conditions rapidly induce a reduction of biomass yield and secondary compounds [3,4]. It has been also shown in a range of MAPs, that these traits are highly variable, also according to the genotype × environment interaction [5,6,7,8].

Shortage of high-quality freshwater, especially if considering the increasing impact of salinity in agriculture, is one of the most limiting factors arising at a global scale. This issue poses dramatic concerns for plant productivity and the achievement of desired quality traits [9,10]. This issue is particularly relevant for MAPs, also because their selling price strongly depends on their phytochemical composition and properties. In particular, water with inappropriate quality strongly alters the ontogenesis, morphology, biochemistry, physiology and metabolic processes of crops [11,12], and in MAPs, it can lead to reduced growth or unexpected quality alteration [13].

Basil (*Ocimum basilicum* L.) is an important warm-season MAP with high potential to be integrated into a wealth of cropping systems [14,15]. The salinity tolerance of basil is considered low [16], and the variability of the responses depends on numerous factors that include the genotype, the growing medium and conditions, including the presence in the soil of microbes with either pathogenic or beneficial activity [16,17,18,19,20,21,22,23,24,25]. Such responses are multidimensional because they affect main parameters of economic importance, such as the leaf fraction on the total above-ground biomass, and the concentration and composition of secondary compounds, including the volatile organic fractions and the essential oil [14,26,27]. Moreover, basil response to salinity is associated with an augmented antioxidant activity and change in the plant morphology, and such responses strongly depend on the salinity and genotypes [23,28,29].

Recently, multilevel selection of plant genotypes, manipulation of environmental conditions and microbial partners have been all invoked to improve tolerance of plants to the stresses and increase plant fitness [30]. In recent years, environmental concerns have strongly increased the scientific interest in the use of free-living microorganisms that, in certain amount and conditions, can be beneficial in agriculture [31]. The activity in the soil and roots of beneficial microbes has proven to directly affect the morphology of plants, also in sub-optimal edaphic conditions [7,19,32,33,34,35,36].

In particular, the inoculation with two fungal biostimulant, namely arbuscular mycorrhizal fungi (AMF) and various *Trichoderma* species, have proven to be able to relieve osmotic stresses, but such effect depended on different mechanisms [37,38], with AMF mostly affecting photosynthetic activity, whereas the influence on Na exclusion and salt-related secondary compounds was variable among the AMF species and plant functional group [39]. In addition, the interaction of Trichoderma with AMF in relieving salt stress seems being dependent on the plant size, and mostly occurring when plant biomass is scarce [40], with scarce interaction between these fungi [41,42] while both fungi can be considered as root elicitors [43].

The microbial inoculum directly influences the plant physiology and accumulation of secondary compounds, irrespective of any potential effect on the plant morphology or uptake of nutrients [33,44]. In sweet basil, different soil microorganisms have been used rhizobacteria to increase the accumulation of essential oil [45] as well as other important phytochemicals, such as phenolic compounds [46]. Such accumulation can also be consequential to a defense priming elicited by the beneficial microbes, especially when from the fungal kingdom, despite its prediction strongly depend on the growing conditions and the plant and microbial genotypes [47]. Moreover, microorganisms such as *Pseudomonas* spp. and *Bacillus lentus* improved the basil response to salt stress [48]. Finally, different works investigated the role of beneficial microorganisms in relation to water stress in basil [49], whereas the interaction of PGRPs with salt stress in modulating the morpho-physiological response and quality of the basil has received very limited attention [50].

Aim of the present work was to study the response of basil subjected to 3 levels of NaCl stress (a low and high stress conditions, compared to a non-stressed control) to the substrate application of a beneficial fungi-based inoculum in term of plant growth, biomass partitioning, physiology (including a targeted analysis of polyphenols and VOCs). The inoculum presence in the root and substrate were also analyzed.

## 2. Results

### 2.1. AMF Root Colonization and Trichoderma spp. in the Substrate

At harvest, we verified the degree of root and substrate colonization by the beneficial fungi applied (Table 1; see also Supplementary Material Table S1 for means ± standard error and statistical analysis, and Supplementary Material Table S2 for the LSmeans). AMF root-colonization was present only for the treated plants. Moreover, saline stress had a strong negative impact on the root colonization by AMF but not for the *Trichoderma* substrate colonization. Even though related to a small sample size, there was a high inverse linear correlation between NaCl concentration the percentage of inoculation (R^2^ = 0.97). On the contrary, the salinity in the NS (and S × I interaction) did not affect the *Trichoderma* presence in soil, which showed a ten-fold increase (from 3.23 Log CFU (g soil)^−1^ to 4.24 Log CFU (g soil^−1^) following inoculation.

### 2.2. Morphological Parameters, Fresh Yield and Dry Biomass Production

In general, all morphological traits were affected by the salinity factor (Table 2; see also Supplementary Material Table S1 for means ± standard error and statistical analysis, and Supplementary Material Table S2 for the LSmeans).

**Figure 1:**
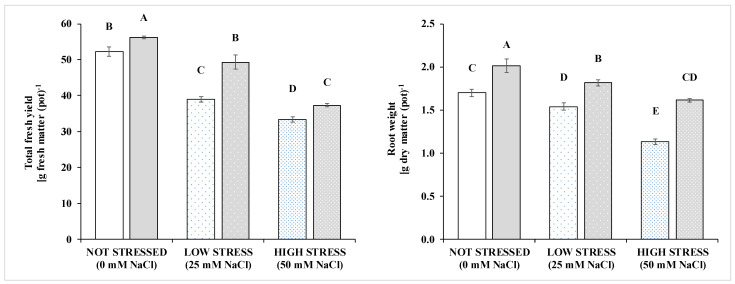
Above ground biomass (fresh weight, left) and root biomass (dry weight, right) of basil grown under non stressed conditions or at two level of salinity stress: low (25 mM of NaCl in the irrigation solution, sparsely dotted bars), and high (50 mM of NaCl in the irrigation solution, densely dotted bars), with (grey bars) or without biostimulant inoculation (white bars). Data are means ± standard error. Bars with a letter in common can’t be considered different at a *p* > 0.05 of the t-grouping of the LSMEANS *p*-differences. See Supplementary Material Tables S1 and S2 for the statistical analysis.

Compared to the non-stressed condition, low-stress salinity reduced these fractions by 16.4–18.6% and high stress by 27.4–29.8%. Salinity showed similar effects on plant height, SPAD, transpiration and leaf water potential. Total fresh and root dry weights varied by the interaction of the salinity and inoculation, with variation by the salinity similar to those observed for the other fractions. Such behavior did not occur for the number of leaves and the leaf dry matter percentage, both of which increased at increasing the salt stress, and some VOCs. Salt stressed plants had a higher number of leaves. An effect on the leaf DM percentage was present only at the highest salt concentration (50 mM NaCl). The biostimulant inoculation had a main effect on most morpho-physiological traits except for the leaf DM percentage, plant height, transpiration and LWP. The inoculation improved the fresh weight more in the low-stressed (+26.6%, Figure 1) than non-stressed (+7.4%) and high-stressed conditions (+12.2%). Whereas inoculation improved root growth more in the high-stress (+42.8%) than in non-stressed (+18.6%) and low stress conditions (17.8%).

In particular, the microbial-based biostimulant increased the total basil biomass, and this was associated with a larger number and size of the leaves. Factors’ interaction was evident only for the R/S and the leaf area of the plants and fresh yield (Figure 1). Specifically, a significantly higher R/S was recorded only for the inoculated plants at the highest saline stress (i.e., 50 Mm NaCl), and biostimulation increased leaf area only under sub-optimal conditions (i.e., at 25 and 50 mM NaCl). Salinity, biostimulation and their interaction also affected the total fresh yield and root dry weight (Figure 1 and Supplementary Material Table S1). As expected, basil yield reduced at increasing salt concentrations, whereas the inoculation of the biostimulant increased yield in all conditions.

### 2.3. Physiological Parameters

The role of the treatments on the net photosynthetic rate and WUEi was similar to those found for the fresh weight. Increasing NaCl concentrations in the nutrient solution from 1 to 50 Mm NaCl caused a reduction in the net photosynthetic rate (Pn) (Table 3), the transpiration rate and Soil Plant Analysis Development (SPAD) index. In addition, there was an increase in the absolute value of the leaf water potential (i.e., it was more negative). Biostimulation had a positive effect on the SPAD index, a non-destructive measure of the chlorophyll content. Moreover, Pn was affected by factors interaction, with the positive effect of the biostimulant evident at 25 mM NaCl. In this condition, the biostimulant increased by 48% the Pn compared to the untread plants, while the biostimulatory effect was negligible in both the control and high saline condition. Despite the significant effect of the NaCl on the Pn, the iWUE was marginally reduced only at 50 mM NaCl and this parameter was neither affected by salt stress or biostimulation. However, it is noteworthy that the higher Pn of the biostimulated plants at 25 mM NaCl significantly improved iWUE, which was similar to that of the plants in the control treatments.

### 2.4. Leaf Mineral Composition

**Figure 2:**
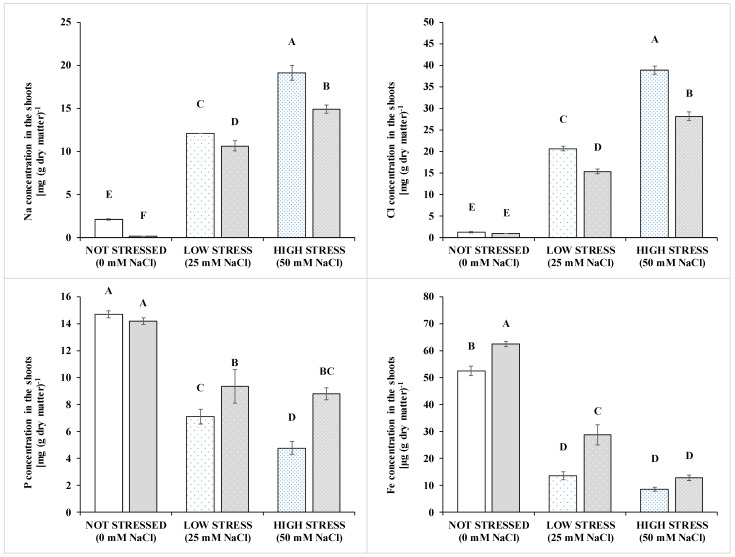
Concentration of **Na** (upper left panel), **Cl** (upper right panel), **P** (lower left panel) and **Fe** (lower right panel) in the shoots of basil grown under non stressed conditions or at two level of salinity stress: low (25 mM of NaCl in the irrigation solution, sparsely dotted bars) and high (50 mM of NaCl in the irrigation solution, densely dotted bars); and inoculated (grey bars) or not (white bars) with a plant-growth promoting inoculum composed of arbuscular mycorrhizal fungi and *Trichoderma koningii*. Data are means ± standard error. Bars with a letter in common can’t be considered different at a *p* > 0.05 of the t-grouping of the LSMEANS *p*-differences. See Supplementary Material Tables S1 and S2 for the complete dataset.

As expected, Na and Cl concentration in leaves increased according to the salinity of the growing medium (Figure 2, Table 4 and Supplementary Material Tables S1 and S2). Biostimulant inoculation had a mitigating effect on Na and Cl accumulation, with significant interaction with the Salinity. The inoculation reduced Cl in leaves by 25.7% and 27.4% in the stressed treatments (25 and 50 mM NaCl, respectively), but not at 1 mM NaCl (Figure 2). Moreover, biostimulation decreased the concentration of the toxic and non-essential Na in all salinity conditions. However, the highest reduction was observed in non-stressed plants (−93.3%) and sweet basil plants under low- and high-stress conditions had a Na concentration reduced by 12.2% and 22.1%, respectively, compared to the non-inoculated conditions.

Increasing salinity caused a significant reduction of the concentration and uptake of the other mineral elements and such a reduction was to a definitely higher extent compared to the biomass. The biostimulant inoculation also had a significant effect on all the minerals in leaves, but with a relevant degree of specificity. Factor interaction was present for P and K among macronutrients, and Fe and Mn among micronutrients. The nitrate concentration in leaves was reduced by both the salinity stress, and the microbial biostimulant with no interaction between the factors. Ca concentration increased in the inoculated plants. Significant S × I interaction was recorded for the other two macroelements, K and P. In particular, the inoculation did not affect the P concentration in the low-stress conditions and improved it by 31.6% and 84.6% in the low-stress and high-stress, respectively. A similar result, but less pronounced in the high stress condition, was found for K concentration.

Among microelements, B concentration was increased by 22.7% in the inoculated compared to not inoculated treatments. Factor interaction was present only for Fe and Mg. Biostimulation increased the concentration of these two elements in leaves in the control condition (+19% and +43%, respectively) and at 25 mM NaCl (+111% and +54%, respectively), whereas differences between treated and untreated plants were not present at 50 mM NaCl. Notably, Fe concentration increased in the inoculated plants by 49% compared to the non-inoculated plants, but such a variation was not detected by the conservative Tukey-Kramer grouping of the *p*-differences (data of the exact *p* not showed). Similar results were found for the Fe and Mn uptakes in the plant.

### 2.5. Antioxidant Activity and Target Polyphenols Profile

**Figure 3:**
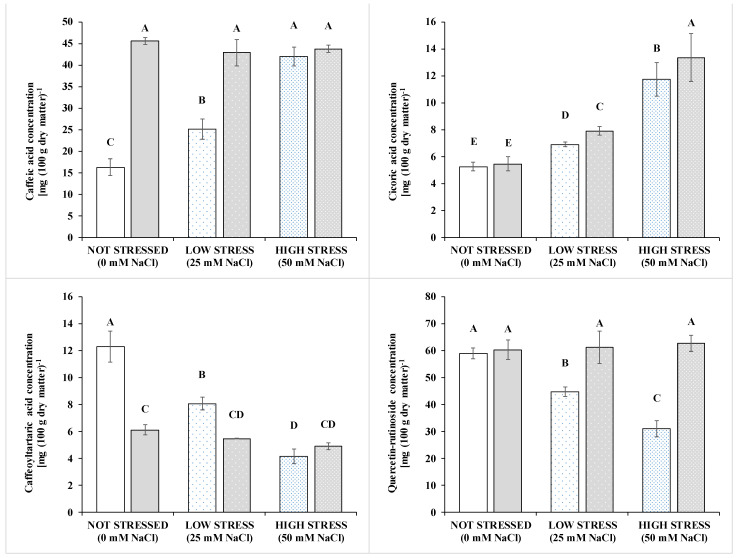
Concentration of polyphenols in the leaves of basil grown under non stressed conditions or at two level of salinity stress: low (25 mM of NaCl in the irrigation solution, sparsely dotted bars) and high (50 mM of NaCl in the irrigation solution, densely dotted bars); and inoculated (grey bars) or not (white bars) with a plant-growth promoting inoculum composed of arbuscular mycorrhizal fungi and *Trichoderma koningii*. Polyphenols are caffeic acid (upper left panel), chicoric acid (upper right panel), and luteolin glycosides (right panel), Caffeoyl-tartaric acid (lower left panel) and Quercetin-rutinoside (lower right panel). HAA and rosmarinic acids behaved similarly as the caffeic acid. Ferulic acids and total polyphenols behaved similarly as the chicoric acids. Data are means ± standard error and expressed per unit dry weight. Bars with a letter in common should not be considered different at a *p* > 0.05 of the t-grouping of the LSMEANS *p*-differences.

The hydrophilic antioxidant capacity (HAA, Table 5) and all targeted polyphenols (except p-coumaric) varied according to a Salinity × biostimulation interaction (Table 5, Figure 3). The salt stress increased HAA by 89.8% in the non-inoculated treatments compared to the non-stressed conditions. The inoculation increased by 52.5% the HAA in the non-stressed condition, with no effect in the stressed treatments.

When averaged over microbial-based biostimulant, the cumaric acid strongly declined from 25 mM to 50 mM salt stress, with unclear differences between 50 mM and 1 mM (the control). The salt stress increased ferulic and rosmarinic acids with different interactions with the biostimulation. The former was highly augmented only at 50 mM NaCl, and only under this condition, there was a remarkable positive effect of the biostimulation (+150% compared to non-inoculated plants). Salinity increased the rosmarinic acid and biostimulation had a positive effect on rosmarinic acid at 1 mM and 25 mM NaCl (+49% and +68%, respectively, compared to the non-inoculated control). Similar differences among treatment were found for the caffeic acid, which increased with salinity especially in the high stress compared to the low stress and control whereas it was increased by the inoculation in the low stress (+71%) and control (+180%) but not in the high stress. The effect of the salinity on the chicoric acid was similar to that found for the rosmarinic acid, whereas the biostimulant increased the chicoric acid under low and high stress (+14% and +45%) but not in the control. An opposite trend compared to these latter acids was recorded for the quercetin-rutinoside (QR), whose concentration diminished in the salt-stressed compared to the control irrespective of the stress degree (either 25 or 50 mM NaCl) and whose concentration was enhanced by the biostimulant in the low and high stress conditions (+30% and +103%, respectively).

### 2.6. Volatile Organic Compounds

The experimental factors never interacted on the relative concentration of each Volatile Organic Compound (VOC) and no effects of either the salinity or the inoculation were seen on the concentrations of β-Pinene, β-Phellandrene, cis-4-Thujanol, 1R-Camphor, α-Bergamotene, γ-Cadinene, α-Terpineol, L-Borneol, Eugenol and the minor constituents referred as “other” in the Supplementary Material Tables S1 and S2. Also, the p-statistic of the salinity for the Eucalyptol was 0.0503. When imposing such a threshold for the computation of the Tukey-Kramer corrected differences, the Eucalyptol fraction on total VOC was 32.6% in the non-stressed treatment, which appeared 3.1% higher than the mean of low and high-salt treatments, with no differences between these treatments. Salitiny and Inoculation only affected (at the threshold of *p* = 0.05), α-Pinene, Hexanal, β-Myrcene, D-Limonene, 2-Hexenal-E, β-Z-Ocimene, 3-Hexen1-ol-Z, 1-Octen-3-ol, β-Linalool, β-Elemene and D-Germacrene.

Differences between 25 mM and 50 mM NaCl were observed only for beta-linalool, which was similar to the control in 25 mM and 22.0% lower than the control in 50 MM. Salt stress irrespective of the solution concentration increased α-Pinene, β-Z-Ocimene and β-Elemene. In addition, D-Germacrene progressively increased at increasing the salinity of the solution. In contrast, Hexanal and 1-Octen-3-ol when the salinity was applied at a similar rate in both salinity levels compared to the control.

Inoculation affected only some minor VOCs, which on the whole contributed to only 7.5% of the VOCs of the non-inoculated plants and 9.1% of the inoculated plants. Inoculation slightly increased α-Pinene, β-Myrcene, D-Limonene and β-Z-Ocimene, and decreased Hexenal-E and Hexen-1-ol-Z. In particular, the presence of the inoculum only affected α-Pinene by 27.4%, β-Myrcene by 34.2%, D-Limonene by 43.7%, β-Z-Ocimene by 30.8%, and reduced 2-Hexenal-E by 42.7% and 3-Hexen1-ol-Z by 21.0%.

The chemotype of the basil under study was characterized by *Eucalyptol* (30.6% ± 2.7%; mean ± standard deviation), β-Linalool (28.2% ± 4.8%), α-Bergamotene (11.0% ± 3.1%) and to a lesser extent by Octen-3-ol (4.8% ± 1.4%).

### 2.7. Multivariate Analyses of the Treatment Role on the Basil Responses

**Figure 4:**
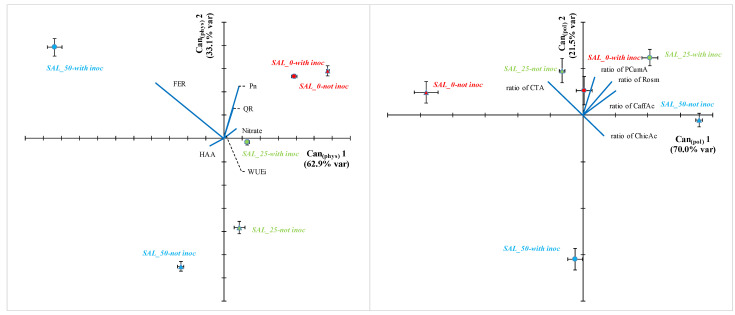
Canonical Discriminant Analysis (CDAs) using standardized data with reciprocal correlation between −0.7 and +0.7 (see Supplementary Material Table S4) of the morphological and physiological parameters (left panel) and polyphenol composition (right panel) in the shoots of basil grown under non stressed conditions (red symbols, indicated as SAL0) or at two level of salinity stress: low (25 mM of NaCl in the irrigation solution, green symbols indicated as SAL25) and high (50 mM of NaCl in the irrigation solution, blue symbols, indicated as SAL50); and inoculated (circles, indicated as Inoc) or not (triangles, indicated as Contr) with a plant-growth promoting inoculum composed of arbuscular mycorrhizal fungi and *Trichoderma koningii*. The CDA of the VOC composition is not shown since it was not significant in depicting the variability (see Supplementary Material Table S4, lines 59–70). Each point is the centroid mean across replicates (±S.E., n = 3) on the canonical axes (CA) 1 and 2. The percentages of the total variance explained by each canonical axis are shown in parentheses. Lines starting from ‘0; 0’ represent the vectors of each determinant (blue and continuous for minerals and black and hatched for bioactive compounds). Determinants for the CDA in the left panel included are: concentration of ferulic acid (FER), concentration of Quercetin-rutinoside (QR), concentration of Nitrate, Net photosynthetic rate (Pn), Hydrophilic antioxidant activity (HAA), Instantaneous water use efficiency (WUEi). Determinants for the CDA in the right panels included rations between each of the polyphenol indicated and the total polypenols: Caffeoyltartaric acid (CTA), Caffeic acid (CaffAc), p-Coumaric acid (pCumA), Chicoric acid (ChicAc), Rosmarinic acid (Rosm). Note that the CDA vectors do not represent perpendicular directions through the space of the original variables. The unit of measure is the same for both axes (2.5 units) in both CDAs.

The measured traits were used for a CDA of the mean effects of the treatments on the plant behavior (Figure 4, Supplementary Material Tables S3 and S4). In addition, the ratio of each polyphenol on the total polyphenols and the ratio of each VOC on the total VOCs were used to perform a CDA of the quality of the product obtained. Notably, the CDA built on the VOC composition failed in retrieving a multivariate structure, and showed a Wilks’ Lambda, Pillai’s Trace, Hotelling-Lawley Trace strongly above 0.05 and similar results were found when pooling together the polyphenol ratios and the VOC in a unique CDA (but see Supplementary Material Table S3)

Three Can _(phys)_ axes were significant for the physiological traits CDA and explained 99.8% of the total variability. After removal of the variables highly correlated each other (while retaining one of them into the model), only six variables were used, including Pn, WUEi, Nitrate, HAA, FER and QR, of which ferulic acid mostly correlated with Can _(phys)_ 1, and ferulic acid, Pn, WUEi and to a lesser extent QR correlated with Can _(phys)_ 2. Notably, salinity clearly separated on the Can _(phys)_ 1 and inoculation on the Can _(phys)_ 2, with increasing separation between inoculated and not inoculated treatments at increasing salinity.

For the composition of the polyphenols, the ratios of the caffeoyltartaric, caffeic, p-cumaric, chicoric and rosmarinic acids on the total polyphenols were retained, of which CTA, caffeic and rosmarinic acids mostly correlating with Can _(pol)_ 1, and p-coumaric acid with Can _(pol)_ 2. However, all the polyphenol ratios similarly determined the total distribution, with Can _(pol)_ 1 and Can _(pol)_ 2 describing the 91.6% of the total variability. In contrast to the physiological traits CDA, the inoculation treatment separated the samples mostly in the Can _(pol)_ 1 of the polyphenol CDA, whereas the salinity unclearly separated in any of the 2 first Can _(pol)_ s.

## 3. Discussion

### 3.1. Role of the Inoculum in the Primary Metabolism, Biomass and Nutrient Accumulation

In general, basil is considered a sensitive species to salt stress [51]. Nonetheless, it showed higher resistance to the NaCl stress compared to other similar species in the Lamiaceae family thanks to its ability to activate catalase, reduce the Na/K ratio in the leaf blades thanks to reduced translocation of Na from the roots and stems to the leaves while moving K to leaf blades [52].

In the present work, Na concentration in the non-stressed plants was 1.1 mg g^−1^, 11.4 mg g^−1^ in the low-stress, 17.0 mg g^−1^in the high-stress. Similarly, Cl concentration in the shoots was 1.1 mg g^−1^, 18.0 mg g^−1^ and 33.5 mg g^−1^in the non-stressed, low stress and high stress conditions, respectively. Thus, increasing salinity from the low to the high stress conditions increased the Na concentration by 49% and Cl concentration by 86%. This suggests that stress increased roughly linearly with increasing the salinity of the solution.

Plant biomass and most of the morphological traits reduced after the application of the salinity stress at a degree similar to both the increases of Na and Cl concentrations. Low-stress salinity reduced these fractions by 16.4–18.6% and high stress by 27.4–29.8% compared to the non-stressed conditions and such degrees of reduction suggest that mild stress occurred in both conditions. In particular, the salinity of 100 mM NaCl (50 mM higher than the high stress in the present study) consisted in reductions of 71–79% of shoot biomass in two genotypes under similar conditions compared to the present study [29]. However, stress by the salinity in term of biomass reductions was found to be extremely variable: similar degrees of salt stress compared to the present study consisted in twice the decrease we observed [24]. Other authors found that a salinity level similar to our high-stress treatment did not consist in a biomass decrease of basil [53]. Differences between the present and other experiments applying similar salinity stress likely arise from both the genotype ability to accumulate Na and thus exclude it form the essential physiological processes, and the availability of K. In particular, Attia et al. [54] showed that a hydroponically grown basil subjected to the same stress degrees as we did can have scarce growth depressions due to the salinity depending on the genotype and its ability to recirculate Na among the plant organs after its uptake. Attia et al. [54] also found that that salinity tolerance is inversely related to leaf number. In turn, leaf number and size can be linked to stomatal density and the ability to modulate the polyphenol concentration and release of hormones [28].

The presence of the inoculum in the soil, and in particular of the arbuscular mycorrhizal symbiosis, exerted a benefit for both the plant growth and nutrient uptake and influenced differentially the secondary metabolites by the degree of salinity stress. In particular, under the low-stress conditions the inoculum increased more shoot than root growth. This likely occurred thank to an increase of the net photosynthetic rate and Instantaneous water use efficiency. Whereas under high stress the inoculum increased definitely more the root than shoot growth. The inoculum used in the present study had various microorganisms, including AMF and *Trichoderma*. Nonetheless, AMF but not *Trichoderma* were absent in the native substrate. Thus, we mostly attribute the results of the application of the inoculum to the AMF rather than the other inoculum components. Similar results were seen in [55], where a similar inoculum increased *Trichoderma* presence more than in the present study. However, according to Singh et al. [56], the differences in *Trichoderma* presence among treatments found here may not be sufficient to ensure a difference in its effect, especially under high nutrient availability, such as in the present, as shown by others [41,57]. Indeed, we cannot exclude that the *Trichoderma* had any effect, since its presence in the soil may have indirectly affected the relationship between ions and the plant; e.g., Colla et al., [40] found an interaction between *Trichoderma* and AMF on 3 contrasting species, all of which, however, yielding only the 18.8% of the above-ground and 15.1% of the below-ground plant biomass compared to the present study. Thus the interaction between *Trichoderma* and AMF in [40] may have been due to both a difference in plant species and the scarce root biomass.

We found that the benefit of the fungi-based inoculum to the plant biomass was on average constant, but such an effect depended on different mechanisms of the plant stimulation at varying the salinity of the growing medium. In particular, the inoculation increased the nitrate concentration and increase the Ca, Mg and B concentrations irrespective of the salt stress degree. In contrast, root biomass increased by 42.8% after the inoculation in the high-stress conditions, and only by 17.8–18.6% in both the low stress and non-stressed conditions. Moreover, the inoculation dramatically increased the net photosynthetic rate and WUEi in the low stress (+48.4% and +49.2% compared to the non-inoculated pots, respectively), but not in the high-stress conditions. At the one time, inoculation increased P concentration dramatically more in the high-stress than the low-stress and an opposite result was found for the Fe concentration. Plants physiological response to salinity has a mutual genetic pattern compared to the response to drought [58,59] and these include the activation of genes related to the C and N metabolism and response to dehydration. However, such responses are strongly genotype-dependent [60]. In basil, such responses also occur after an increase in the leaf Na accumulation [29]. The basil genotype use here showed a strong ability to respond to various degrees of salt stress, including a 2-fold (100 mM) and 4-fold (200 mM) stress if compared to the present study [29,61]. We showed here that the activity of the beneficial microbes inoculated consisted in a strong increase in the leaf number and leaf area irrespective of the salt stress, and this may have contributed to increase the plant ability to withdraw Na in the leaves and withstand the salt stress. However, the inoculum stimulation to the leaf area mostly occurred in low stress (25 mM) condition rather than the high (50 mM) stress. Such a result was likely due to both a high ability of the genotype used here to emit new leaves under salt stress [28] and high C investment of the plant in the root growth in the high-stress conditions, so that photosynthates were less available for the leaf emission. This also agrees with the higher inoculum stimulation of the Fe and Mn uptake in the low-stress conditions, which may have further stimulated the leaf emission. Indeed, we also found a higher inoculum stimulation of the P uptake in the high than low stress conditions. This may have been due to both a lower P availability for the plants due to the high salt concentration in the soil solution and plant tendency to invest C in the root system after a P starvation [62]. Nonetheless, we found that the interaction of the inoculation with salt stress in the present study in term of Fe concentration and uptake (F = 4.32; *p* = 0.044, and F = 6.37, *p* = 0.016, respectively) was scarce. This occurred despite the fact that at these thresholds the factors are considered to change “significantly”, and differences among treatments were mostly due to the presence of the inoculum and seen by a magnitude of the effect (+156% of Fe Uptake in the low stress and +53% in the high stress). In particular, AMF showed to be able to amply sustain the uptake of Fe-bound P by the plant [32,63] and some *Trichoderma* species showed to sustain P or Fe uptake by a siderophore production [64,65,66,67]. The role of the inoculum in the exclusion of the Na uptake in the high stress was higher than in the low stress. Thus, on the whole, the benefit of the inoculum to the plant under the stress conditions appeared to be mediated by a stimulation of the plant activity in the low stress and stimulation of the stress avoidance system in the high stress. Such result agrees with earlier reports of the AMF effects on salt stress of basil with variable salt tolerance subjected to similar Na concentration compared to the present study [19]. In addition, *Trichoderma* may have contributed to a Na exclusion for the plant [68,69,70]. This result was confirmed by the CDA, especially if considering that in other experiments conducted under a high N, P or Zn conditions [71,72], such as in the present study, high nutrient availability reduced the AMF benefit in the plant in the K, Fe, Mn and Zn. Indeed, we cannot exclude that the stimulation of the P uptake by the inoculum was also mediated by a higher release of Ca and other divalent cations in the soil solution, which may have further reduced the P availability due to phosphate insolubilization. Indeed, [19] showed that an increase of the salinity due to Ca may consist in a competition between the Ca and other divalent and trivalent cations, whose uptake was stimulated here, and such result was not seen after an increase of the salinity due to Na. Furthermore, earlier reports showed that AMF alters the K:Na or Ca:Na ratio in its biomass compared to the growing medium [73] and can consist in a similar response in the plant leaf [74], thus acting as a barrier to Na uptake.

### 3.2. Role of the Inoculum on the Plant Quality

We expected to find an increase in the phenolic and other antioxidant compounds contents after the salt stress application, since it appears to be a conserved response to salt concentration, including in basil [21,75]. Indeed, we found that both the salt stress levels applied here dramatically stimulated the hydrophilic antioxidant activity of the plants, but that such a stimulation also occurred in the unstressed conditions at the presence of the beneficial inoculum. Such result also occurred under various water availability [55], whereas others found a lesser stimulation of the carotene content in the control conditions compared to the salt stress after AMF inoculation [21]. At the one time, inoculation stimulated total polyphenol content in all conditions, although at variable rates (+23% in the non-stressed, +29% in the low stress and +63% in the high-stress conditions). These results indicate that the inoculum contributed to a direct stimulation of the plant mechanisms of the stress tolerance, in addition to its effect on the plant nutrition and photosynthetic activity. The effects of the inoculum on each phenolic compound concentration were clearly seen to be similar to the effect on the total phenolic concentration, and especially on chicoric acid and quercetin-rutinoside. The analysis of the incidence of each phenolic compound on the total phenolic compounds clearly indicated that the inoculum strongly affected the phenolic composition. On the one hand, the inoculum reduced the caffeoyl-tartaric acid contribution to the total phenolics in all conditions, especially in the control pots; on the other hand, it increased caffeic acid and rosmarinic acid contributions to the total phenolics in the control pots and reduced it in the high-stress conditions, with intermediate results in the low stress. An opposite result was found for the quercetin-rutinoside contribution to the total phenols. Similar results were found for luteolin, but not for the quercetin bearing compounds after a drought application [55]. Hazzoumi et al. [75] showed that AMF increased the total phenolic compound contents in the non-stressed but not in the drought-stressed conditions. The variation of these phenolic components in basil subjected to salt stress may depend on the genotypic response to salinity, as shown by [20], the latter of which also reported that AMF may differentially stimulate the accumulation of each of these compounds, reporting similar results compared to the present study [76]. The latter authors also highlighted that the relative concentration of these compounds may be strongly related to the Ca concentration. This result agrees with our previous work when under contrasting water availability [55] and with the present results. In addition, it was shown that AMF can stimulate the rosmarinic and caffeic acids concentration irrespective of their effect on the P nutrition of the plants [46].

In contrast to the main phenolic compounds, salt stress scarcely affects the essential oil composition and no effect of the inoculum was found. Khalediyan et al. [77] showed scarce effects of the inoculation with AMF on the accumulation of single EO compounds in basil. Other works showed that the AMF effects on the EO composition of various *Ocimum* species are variable and may depend on the plant genotype, AMF genotype and EO compound taken into account [78,79,80,81,82] and indeed, in the present study, the CDA procedure failed in retrieving mean effects of the treatments applied on the EO composition. This suggests that basil biosynthesis of the EO has strong genetic control. Nonetheless, it has been shown that *Trichoderma harzianum* may induce EO accumulation through direct intervention of its metabolites on the EO biosynthetic pathways, despite such an effect depending on the *Ocymum* species [83]. These effects may have been due, in our experiment, to a direct effect of both fungi on the phenylpropanoid, mevalonate, carotenoid and oxylipins pathway, as seen in tomato and other species when inoculated with AMF, *Trichoderma* or beneficial bacteria [84,85,86,87] so that at the one time the plant antioxidant status was relieved by the presence and activity of the microbial partner and, at the other time, such pathways may have been directly affected by some microbial metabolite.

## 4. Materials and Methods

### 4.1. Biological Material and Experimental Design

The experiment was carried out in 2014 (from May to July) in a glasshouse at the experimental farm ‘Torre Lama’ (Bellizzi, SA, Italy) of the Department of Agricultural Sciences (University of Naples Federico II). Seeds of a Genovese-type sweet basil (*Ocimum basilicum* L. ‘Gecom’), obtained from “Società Agricola Italiana Sementi” (Cesena, FC, Italy), were sown in vermiculite. Three-true leaves plantlets were transplanted to black plastic pots, with approximately 1.3 L of a Brill 3 peat, at a 23 plants per square meter density. Mean air temperature during the trial ranged from a minimum of 18 °C to a maximum of 34 °C, while relative humidity ranged from 54% (min) to 79% (max).

Plants grew in an open loop hydroponics system (i.e., a fresh nutrient solution [NS] was introduced at each irrigation treatment using a drip system). The base NS, in de-ionized water, had the following composition: 13.0 mM N-NO_3_^−^; 1.0 mM N-NH_4_^+^; 1.75 mM S; 1.5 mM P; 5.0 mM K; 4.5 mM Ca; 2.0 mM Mg; 20 µM Fe; 9.0 µM Mn; 0.3 µM Cu; 1.6 µM Zn; 20.0 µM B; 0.3 µM Mo. The NS was pumped from independent tanks at a 2 L h^−1^ flow rate. The trial was conducted with a full factorial design, with biostimulation and salt stress as variable factors. The plant treatment with the biostimulant had two levels, not inoculated [referred as “not inoc”] or inoculated [referred as “with inoc”]. Biostimulation was applied at transplant placing one tablet per pot just below the basil roots. The microbial-based biostimulant tablet (4.5 g) (Asir Horto, Agrotecnologías Naturales S.L., Tarragona, Spain) contained 225 spores of arbuscular mycorrhizal fungi (AMF) per tablet including *Rhizoglomus irregulare* (BEG72) and *Funneliformis mosseae* (BEG234), each at the dose of 25 spores g^−1^. In addition, the tablet contained the inoculum of the fungus *Trichoderma koningii* TK7 strain at 1 × 10^6^ UFC g^−1^; and the bacteria *Bacillus megaterium* MHBM77 1 × 10⁶ UFC g^−1^ and *B. megaterium* MHBM06 1 × 10⁶ UFC g^−1^. Each tablet also contained N:P_2_O_5_:K_2_O at rates of 8:5:4 and was composed by the 50% of organic material, according to the producer’s indications.

The salt stress has three levels, namely: basal salt concentration (0 mM NaCl, also referred as “Control”), low salt stress (25 mM NaCl) and high salt stress (50 mM NaCl). The three salinity levels were obtained adding NaCl adding NaCl to the control NS to reach 25 mM for the low salt level, and 50 mM for the high salt level. The pH of the NS was 6.0 while the electrical conductivity (EC) was 2.1 dS m^−1^ for the NS with the basal salt level, 4.4 dS m^−1^ for the low salt treatment and 6.7 dS m^−1^ were for the high salt level. The irrigation with the low and high salt NSs started eight days after transplant (DAT).

Experimental units (each made of 15 plants) were replicated three times (for a total of 270 plants) and arranged in a randomized complete block design.

### 4.2. Biometric Measurements and Leaf Analyses

In this case, 44 DAT, when plants were at pre-flowering stage, the leaves, the stem and the root from ten plants per experimental unit were harvested, weighted (fresh weight) and dried at 70 °C for 3 d until constant weight (dry weight), using an analytical balance (Denver Instruments, Denver, Colorado, USA). The total leaf area per plant (n = 10) was measured with a portable leaf area meter (Li-Cor3000, Li-Cor, Lincoln, NE, USA). Oven-dried leaf tissues were used for the determination of the mineral profile as described [11] using an inductively coupled plasma emission spectrophotometer (ICP Iris, ThermoOptek, Milan, Italy). Before harvest, the SPAD index was also measured on a total of 20 fully expanded leaves per experimental unit with a portable chlorophyll meter (SPAD-502, Konica Minolta, Tokyo, Japan). A WP4 dew point potentiometer (Decagon Devices, Pullman, WA) was used to measure the leaf water potential. The net photosynthetic rate (P_n_) and transpiration rate (T_r_) were measured with a portable gas exchange analyzer equipped with a 6.25 cm^2^ cuvette window area (LCA-4; ADC BioScientific Ltd., Hoddesdon, UK). Measurements on six fully expanded leaves per treatment were carried out within a 2 h interval across solar noon. Photosynthetic photon flux density, relative humidity and carbon dioxide concentration were set at 665 ± 15 μmol m^−2^ s^−1^, 55 ± 1.2 % and 329 ± 0.5 ppm, respectively. The flow rate of air was 400 µmol s^−1^. The intrinsic water use efficiency was calculated as P_n_/T_r_.

### 4.3. Hydrophilic Antioxidant Activity and Quantification of Target Polyphenols

Fresh sweet basil leaf samples from three plants per experimental plot were frozen in liquid nitrogen, stored at −80 °C and lyophilized in an Alpha 1-4 LSC plus (Osterode, Germany) freeze drier. The hydrophilic antioxidant activity (HAA) was assessed as reported Fogliano et al. [88] using the *N*,*N*-dimethyl-p-phenylenediamine method and reading samples at 505 nm with a DR 2000 spectrophotometer (Hach Co., Loveland, Colorado, USA). HAA was expressed as mmol ascorbic acid per 100 g of dry weight.

The phenolic extracts from leaves were obtained as previously described [89]. Analyses were carried out in triplicates. Briefly, 500 mg of dried leaf samples were added to five mL of 70% methanol in water, thoroughly mixed for 1 min, sonicated for 30 min, centrifuged (14,800 rpm for 10 min) and filtered through paper (Whatman). Concentration of caffeic acid, caffeil-tartaric acid, chicoric acid, p-coumaric acid, ferulic acid, quercetin-rutinoside, rosmarinic acid was determined by LC/MS/MS. Compounds were separated with a HPLC apparatus equipped with two micropumps, Perkin-Elmer Series 200 (Norwalk, CT, USA) and a Prodigy ODS3 100Å column (250 mm × 4.6 mm, particle size 5 µm) (Phenomenex, CA, USA) using as eluents water 0.2% formic acid (A) and acetonitrile/methanol (60:40, *v/v*) (B). The gradient program was: 20–30% B (6 min), 30–40% B (10 min), 40–50% B (8 min), 50–90% B (8 min), 90–90% B (3min), 90–20% B (3min), at a constant flow rate of 0.8 mL min^−1^. Injection volume was of 20 µL. MS and MS/MS analyses of extracts were performed on a triple quadrupole mass spectrometer (API 3000, Applied Biosystems, Canada) equipped with an electrospray ion source in the negative ion mode. Molecular weight, fragmentation pattern, comparative retention time and UV absorption were employed for Information-Dependent Acquisition mode of data collection. Precursor ion and the MS/MS product ions of the phenolic compounds retrieved in the extracts are expressed as “Compound: *m/z* of the molecular ion in [M-H]-; and *m/z* of the fragment(s) separated by a comma” as follows. Caffeic acid: 179; 135. Caffeil-tartaric acid: 311; 179. Chicoric acid: 472; 309, 291, 179. p-Coumaric acid: 163; 119, 113. Ferulic acid: 193; 134, 178, 149. Quercetin-rutinoside: 609; 301. Rosmarinic acid: 359; 197, 179, 161.

### 4.4. Arbuscular Mycorrhizal Fungi Root Colonization and Quantification of Trichoderma

The AMF root colonization was examined at harvest. Briefly, root samples were washed with 10% KOH at room temperature and stained with 0.05% trypan blue in lactophenol as previously reported [90]. The quantification of the AMF colonization was performed calculating the percentage of root segments containing arbuscules + vesicles using a gridline intercept method [91]. Quantification of *Trichoderma* spp. was performed by serial plating soil dilution on a *Trichoderma*-selective agar (TSA) medium [92]. In brief, 10 g of root-substrate was suspended in sterile distilled water to give a 1:10 dilution and then serially diluted up to 1:10^8^. Aliquots (10 µL) of each dilution were plated on Petri dishes with TSA (four replicates per dilution) and incubated for 72 h. Colonies of *Trichoderma* spp. were visually counted and expressed as CFU per g of dry soil.

### 4.5. Analysis of Volatile Compounds

The analysis of Volatile Compounds, starting from 2 g of freeze-dried leaf material, was performed essentially as previously described [93] using a solid-phase microextraction (SMPE) sampling technique coupled with gas chromatography/mass spectrometry (GC/MS). Compounds were identified considering their retention indices and mass spectra according to the NIST Atomic Spectra Database (similarity values: 85–100%). Relative quantification was carried out based on peak area.

### 4.6. Computation and Statistical Analysis

Data of *Trichoderma* CFU were log-transformed before analysis. A two-way analysis of variance was performed by means of a general linear mixed model (Glimmix procedure) in SAS/STAT 9.2 software (SAS Institute Inc., Cary, NC, USA) according to the experimental design. Restricted maximum likelihood (REML) was used to produce unbiased estimates of variance and covariance parameters. This procedure is capable of modelling non-normal data and correcting for heteroscedasticity [94]. Block was treated as a random factor. Differences among means were compared by applying t-grouping at the 5% probability level to the LSMEANS *p*-differences.

A correlation among all traits was performed by means of the CORR procedure in SAS/STAT 9.2. Data on concentration of minerals (including nitrate) and target phenolic acids were used for a Canonical discriminant analysis (CDA, Candisc procedure in SAS/STAT 9.2) on non-highly correlated variables. To select variables to be included in the CDA, when two or more variables were highly correlated (|r| > 0.70), one was discarded to avoid element weighting distortion as suggested by Pengelly and Maass [95]. To run the CDA, data were standardized to a mean equal to 0 and standard deviation equal to 1 to avoid a distortion of the analyses by the variable ranges and units of measurements. Standardized raw data were used as vectors to summarize among-treatments variation. When the Wilks’ Lambda, Pillai’s Trace and Hotelling-Lawley Trace were lower than 0.05, thus suggesting the presence of a multivariate structure, treatments on the CDA were separated by computing the probability that their distance on the hyperspace composed by only the canonical axes was higher than the Mahalanobis distance at a *p* < 0.05. Only the canonical axes whose variability explained was significantly higher than zero were considered.

## 5. Conclusions

The present research focused on the development of innovative and eco-friendly approaches such as microbial-based biostimulant for enhancing crop yields and functional quality of the produce under optimal and sub-optimal conditions. We mostly attributed the benefit of the inoculum to the AMF rather than the *Trichoderma* due to both the absence of the AMF in the control, the presence of native *Trichoderma* in the substrate. The inoculum clearly sustained the yield and quality components of the basil, with no effects on the volatile organic compound composition and stronger effects on yield and polyphenols, which contribute to the antioxidant activity and can increase the shelf life of the product. The benefit of the inoculum in term of yields was evident under all salinity conditions but depended on different pathways. In the unstressed control and low stress (25 mM) it was mostly mediated by a direct effect on the plant photosynthetic rate and intrinsic water use efficiency, both of which due to a both a better nutritional status and a direct inoculum effect. In the high stress conditions, it mostly depended on the inoculum ability to sequester Na and sustain the plant uptake of P. Notably, the inoculum reduced the nitrate concentration. Nonetheless, it is unlikely that basil or its preparation (e.g., the pesto sauce in Italy) can massively contribute to nitrate load in the human diet. These results have implication for the use of irrigation water with relatively high salt concentration in controlled conditions and the role on the composition of the polyphenols or on the relative concentration on some single VOCs highlight the potential of the beneficial inocula in favoring the obtainment of given quality traits.

## Figures and Tables

**Table 1:** Effect on the salt stress (**S**), biostimulant inoculation (**I**) and their interaction (**S × I**) on the beneficial microorganisms. Data are means ± standard error. *p*-values lower than 0.05, and related F-values, are in bold. Within each column, means with a letter in common can’t be considered different at a *p* > 0.05 of the t-grouping of the LSMEANS *p*-differences. When the salt stress but not the S × I was significant (threshold *p* = 0.05), letters denoted mean differences among salinity levels. When I but not the S × I was significant (threshold *p* = 0.05), letters are not displayed since the inoculum levels were two. See Supplementary Material Tables S1 and S2 for the complete dataset. **n.a.** denotes not available test.

		Root AMF Colonization		Trichoderma Substrate Colonization	
		AMF		Tricho	
Salinity (mM NaCl)	Biostimulant	% root colonised		Log CFU g^−1^ soil	
0	not inoc			A	3.28 ± 0.06
	with inoc	44.9 ± 3.54			4.32 ± 0.04
25	not inoc			B	3.2 ± 0.08
	with inoc	27.93 ± 1.39			4.25 ± 0.03
50	not inoc			C	3.22 ± 0.11
	with inoc	17.8 ± 1.55			4.16 ± 0.06
		F	*p*	F	*p*
Salinity (S)		41.4	0.002	1.9	0.195
Inoculation (I)		n.a.	n.a.	485.7	<0.001
S × I		n.a.	n.a.	0.6	0.569

**Table 2:** Effect on the salt stress (**S**), biostimulant inoculation (**I**) and their interaction (**S × I**) on the plant morphological parameters. Data are means ± standard error. *p* values lower than 0.05, and related F-values, are in bold. Within each column, means with a letter in common can’t be considered different at a *p* > 0.05 of the t-grouping of the LSMEANS p-differences. When S but not the S × I was significant (threshold *p* = 0.05), letters denoted mean differences among salinity levels. When I but not the S × I was significant (threshold *p* = 0.05), letters are not displayed since the inoculum levels were two. See Supplementary Material Tables S1 and S2 for the complete dataset.

		Shoot DW				Total Biomass				Root to Shoot Ratio				Number of Leaves				Leaf DM Percentage					Leaf Area		
		S_DW_				TotBiom_DW_				R/S				N_L_				L_DM%_					LA		
Salinity (mM NaCl)	Biostimulant	g pot^−1^				g pot^−1^				g g^−1^				n				%					cm^2^ plant^−1^		
0	not inoc		7.33 ± 0.3		A		9.03 ± 0.33		A		0.23 ± 0.01		CD		103.3 ± 1.8		B		8.62 ± 0.16		B		1754 ± 41		A
	with inoc		7.85 ± 0.41				9.86 ± 0.43				0.26 ± 0.01		CB		121.8 ± 0.9				8.91 ± 0.64				1852 ± 22		A
25	not inoc		5.64 ± 0.02		B		7.18 ± 0.06		B		0.27 ± 0.01		AB		108 ± 2.5		AB		8.91 ± 0.07		B		1205 ± 45		C
	with inoc		6.79 ± 0.19				8.6 ± 0.17				0.27 ± 0.01		AB		126.5 ± 5.1				8.81 ± 0.37				1569 ± 54		B
50	not inoc		5.38 ± 0.05		C		6.51 ± 0.07		C		0.21 ± 0		D		116.6 ± 1.1		A		10.48 ± 0.37		A		1013 ± 17		D
	with inoc		5.5 ± 0.24				7.12 ± 0.18				0.29 ± 0.01		A		127.1 ± 1.7				9.14 ± 0.35				1166 ± 31		C
		F		*p*		F		*p*		F		*p*		F		*p*		F		*p*		F		*p*	
Salinity (S)		42.5		<0.001		57.8		<0.001		3.9		0.057		6.4		0.016		4.8		0.035		184.4		<0.001	
Inoculation (I)		9.6		0.011		22.6		<0.001		20.4		0.001		56.4		<0.001		1.6		0.235		45.4		<0.001	
S × I		2.4		0.142		1.5		0.279		11.4		0.003		1.6		0.255		2.6		0.125		7.1		0.012	

**Table 3:** Effect on the salt stress (**S**), biostimulant inoculation (**I**) and their interaction (**S × I**) on the physiological paramters. Data are means ± standard error. *p* values lower than 0.05, and related F-values, are in bold. Within each column, means with a letter in common can’t be considered different at a *p* > 0.05 of the t-grouping of the LSMEANS *p*-differences. When S but not the S × I was significant (threshold *p* = 0.05), letters denoted mean differences among salinity levels. When I but not the S × I was significant (threshold *p* = 0.05), letters are not displayed since the inoculum levels were two. See Supplementary Material Tables S1 and S2 for the complete dataset.

		Net Photosynthetic Rate				Transpiration				Instantaneous Water use Efficiency				Leaf Water Potential				SPAD Index			
		Pn				Tr				WUEi				LWP				SPAD			
Salinity (mM NaCl)	Biostimulant	μmol CO_2_ m^−2^ s^−1^				μmol H_2_O m^−2^ s^−1^				μmol CO_2_ (mmol H_2_O)^−1^				MPa				SPAD units			
0	not inoc		16.43 ± 0.68		A		4.8 ± 0.17		A		3.44 ± 0.23		AB		−0.48 ± 0.06		A		40.26 ± 1.18		A
	inoc		17.04 ± 0.8		A		4.66 ± 0.42				3.69 ± 0.25		A		−0.59 ± 0.09				41.46 ± 0.51		
25	not inoc		9.35 ± 0.15		C		3.69 ± 0.28		B		2.56 ± 0.17		C		−0.88 ± 0.09		B		36.66 ± 0.82		B
	inoc		13.88 ± 0.53		B		3.64 ± 0.15				3.82 ± 0.13		A		−0.79 ± 0.13				37.92 ± 1.29		
50	not inoc		7.94 ± 0.31		C		2.56 ± 0.28		C		3.15 ± 0.24		BC		−1.2 ± 0.09		C		33.59 ± 0.52		B
	inoc		9.77 ± 0.86		C		3.46 ± 0.17				2.86 ± 0.38		BC		−1.13 ± 0.1				36.91 ± 1.4		
		F		*p*		F		*p*		F		*p*		F		*p*		F		*p*	
Salinity (S)		86.1		<0.001		22.1		<0.001		2.7		0.115		21.7		<0.001		15.5		<0.001	
Inoculation (I)		21.8		<0.001		1.2		0.296		4.2		0.069		0.1		0.813		5.3		0.043	
S × I		5.4		0.026		2.4		0.141		5.1		0.029		0.6		0.555		0.7		0.518	

**Table 4:** Effect on the salt stress (**S**), biostimulant inoculation (**I**) and their interaction (**S × I**) on the mineral concentration in leaves. Data are means ± standard error. *p* values lower than 0.05, and related F-values, are in bold. Within each column, means with a letter in common can’t be considered different at a *p* > 0.05 of the t-grouping of the LSMEANS *p*-differences. When S but not the S × I was significant (threshold *p* = 0.05), letters denoted mean differences among salinity levels. When I but not the S × I was significant (threshold *p* = 0.05), letters are not displayed since the inoculum levels were two See Supplementary Material Tables S1 and S2 for the complete dataset.

**Salinity (mM NaCl)**	**Biostimulant**	**Nitrate**				**P**				**K**				**Ca**				**Mg**			
		**mg (g FW)^−1^**				**mg (g DW)^−1^**				**mg (g DW)^−1^**				**mg (g DW)^−1^**				**µg (g DW)^−1^**			
0	not inoc		3.08 ± 0.13		A		14.7 ± 0.24		A		37.96 ± 1.31		A		12.89 ± 0.68		A		4.41 ± 0.05		A
	with inoc		2.64 ± 0.22				14.16 ± 0.25		A		38.89 ± 1.64		A		13.72 ± 1.21				4.72 ± 0.37		
25	not inoc		2.82 ± 0.03		B		7.09 ± 0.53		C		21.44 ± 0.51		D		6.41 ± 0.35		B		1.52 ± 0.15		B
	with inoc		2.31 ± 0.06				9.32 ± 1.24		B		29.41 ± 0.53		B		10.1 ± 0.42				1.96 ± 0.09		
50	not inoc		2.38 ± 0.09		B		4.77 ± 0.45		D		20.5 ± 0.41		D		5.74 ± 0.2		B		0.83 ± 0.03		C
	with inoc		2.24 ± 0.08				8.78 ± 0.46		BC		24.67 ± 0.97		C		9.13 ± 0.31				1.29 ± 0.02		
		F		*p*		F		*p*		F		*p*		F		*p*		F		*p*	
Salinity (S)		10.6		0.003		84.1		<0.001		141.1		<0.001		51.2		<0.001		240.8		<0.001	
Inoculation (I)		13.7		0.004		13.8		0.004		28.2		<0.001		26.5		<0.001		8.5		0.016	
S × I		1.4		0.303		6.7		0.014		6.1		0.018		3.1		0.088		0.1		0.888	
**Salinity (mM NaCl)**	**Biostimulant**	**Na**				**Cl**				**Fe**				**B**				**Mn**			
		**mg (g DW)^−1^**				**mg (g DW)^−1^**				**µg (g DW)^−1^**				**µg (g DW)^−1^**				**µg (g DW)^−1^**			
1	not inoc		2.12 ± 0.08		E		1.27 ± 0.15		E		52.45 ± 1.64		B		43.6 ± 5.09		A		25.83 ± 2.72		B
	with inoc		0.14 ± 0.01		F		0.91 ± 0.04		E		62.32 ± 1		A		47.43 ± 1.91				38.33 ± 0.52		A
25	not inoc		12.1 ± 0.02		C		20.67 ± 0.59		C		13.56 ± 1.5		D		17.41 ± 1.04		B		10.81 ± 0.59		D
	with inoc		10.63 ± 0.59		D		15.34 ± 0.57		D		28.66 ± 3.76		C		28.36 ± 0.85				16.68 ± 0.59		C
50	not inoc		19.12 ± 0.84		A		38.79 ± 0.92		A		8.49 ± 0.67		D		14.46 ± 1.5		C		7.18 ± 0.04		D
	with inoc		14.9 ± 0.47		B		28.16 ± 1.07		B		12.65 ± 0.99		D		16.82 ± 0.59				9.09 ± 0.21		D
		F		*p*		F		*p*		F		*p*		F		*p*		F		*p*	
Salinity (S)		611		<0.001		1163.6		<0.001		348.2		<0.001		85.8		<0.001		224.1		<0.001	
Inoculation (I)		46.2		<0.001		98.4		<0.001		40.8		<0.001		8.7		0.015		49		<0.001	
S × I		5		0.031		29.3		<0.001		4.3		0.045		1.9		0.206		10.2		0.004	

**Table 5:** Results of the statistical analysis and mean effects of the treatments for the hydrophilic antioxidant activity and polyphenol in basil subjected to irrigation at increasing salinity and inoculated or not with a fungal based inoculum comprising arbuscular mycorrhizal fungi and *Trichoderma koningii*. See Supplementary Material Tables S1 and S2 for the complete dataset. The analysis was run with a general mineral mixed model with restricted maximum likelihood (REML) to produce unbiased estimates of variance and covariance parameters. F and *p* values lower than 0.05 are shown in bold. Treatments were salinity of the irrigation (**S**) and microbial-based biostimulant inoculation (**S**). Data are means ± standard error. When the salinity but not the S × I interaction were significant (threshold *p* = 0.05), letters denoted mean differences among salinity levels. In particular, within each column, means with a letter in common can’t be considered different at a *p* > 0.05 of the t-grouping of the LSMEANS *p*-differences. When inoculum but not the S × I interaction was significant (threshold *p* = 0.05), no letters were displayed since the inoculum statistical levels were only 2.

Salinity (mM NaCl)	Biostimulant	Hydrophilic Antioxidant Activity				*p*-Coumaric Acid				Ferulic Acid				Rosmarinic Acid			
		HAA				pCumAc				FerAc				RosmAc			
		mmol Ascorbic Acid eq. (100 g DW)^−1^				mg (100g DW)^−1^				mg (100g DW)^−1^				mg (100g DW)^−1^			
0	not inoc		22.2 ± 1.09		C		7.57 ± 0.39		AB		32.89 ± 0.22		C		11.77 ± 0.2		C
	with inoc		33.86 ± 3.36		B		7.97 ± 1.21				34.85 ± 0.38		C		16.53 ± 2.34		B
25	not inoc		42.09 ± 1.66		A		8.58 ± 0.7		A		35.09 ± 1.08		C		17.61 ± 2.06		B
	with inoc		45.76 ± 0.99		A		9.5± 0.74				36.11 ± 0.58		C		26.38 ± 2.17		A
50	not inoc		42.18 ± 1.59		A		6.23 ± 0.87		B		44.8 ± 2.23		B		26.72 ± 2.75		A
	with inoc		43.84 ± 0.42		A		6.25 ± 0.44				113.56 ± 3.68		A		25.33 ± 1.8		A
		F stat.		*p*-value		F stat.		*p*-value		F stat.		*p*-value		F stat.		*p*-value	
Salinity (S)		82.1		<0.0001		6.6		0.015		516.8		<0.001		38.8		<0.0001	
Inoculation (I)		24.8		<0.001		0.5		0.498		284		<0.001		31.8		0	
S × I		7.2		0.012		0.2		0.842		248.1		<0.001		7.8		0.009	

## Data Availability

The datasets generated for this study are available on request to the corresponding author.

## Supplementary Materials

The following are available online at https://www.mdpi.com/article/10.3390/pathogens10070797/s1, Table S1. Arithmetic means (n = 3) and results the statistical analysis and mean effects of the treatments for morphological and physiological parameters in basil subjected to irrigation at increasing salinity (1, 25 and 50 mM) and inoculated or not with a fungal based inoculum comprising arbuscular mycorrhizal fungi and *Trichoderma koningii*. Table S2. Least square means and results the statistical analysis and mean effects of the treatments for morphological and physiological parameters in basil subjected to irrigation at increasing salinity (1, 25 and 50 mM, indicated as “SAL”) and inoculated or not with a fungal based inoculum (indicated as “INOC”) comprising arbuscular mycorrhizal fungi and *Trichoderma koningii*. The folder also provide a tool for a rapid surfing among results by using the filter tools on the coloumn “SIGN”. In particular, when an interaction at *p* < 0.05 between salinity and inoculation occurred, the variable was labeled as “INT” irrespective of the main effects. When p of only salinity or only inoculation, but not the other treatment was lower than the conventional threshold of 0.05, the variable was labeled as SAL or INOC, respectively. When both main factors but not their interaction showed a *p* < 0.05, the variable was labeled as “BOTH”. When none of the main factors or their interaction showed a *p* < 0.05, the variable was labeled as “NONE”. Table S3. Results for the canonical discriminant analyses built with physiological data (indicated as “Phys”), with polyphenol composition in term of the ratio between each polyphenol and the total polyphenols (indicated as “Ratios”), with Volatile Organic Compound (indicated as “VOC”) or both the polyphenol composition and VOC (indicated as “R&V”). Multivariate Statistics and F Approximations are shown. Table S4. Correlations among pair of variables used in the present study along with a tool for a easier visualization of correlations whose absolute value (modulus) is higher than the fixed threshold.
